# Maternal Immune System and State of Inflammation Dictate the Fate and Severity of Disease in Preeclampsia

**DOI:** 10.1155/2021/9947884

**Published:** 2021-06-05

**Authors:** Xin Zhao, Shuying Chen, Cui Zhao, Fei Xia

**Affiliations:** ^1^Department of Gynecology and Obstetrics, The First Affiliated Hospital of Soochow University, Soochow, China; ^2^Department of Gynecology and Obstetrics, Affiliated Hospital of Xuzhou Medical University, Xuzhou, China

## Abstract

Preeclampsia, a multisystem disorder in pregnant women, is diagnosed by onset of new hypertension, proteinuria, or organ damage. Antiangiogenic factors, such as soluble fms-like tyrosine kinase 1 (sFlt1) and soluble endoglin (sEng), are long known to be involved in preeclampsia. However, the role of maternal immune system and inflammation in promotion of preeclampsia has lately been a subject of immense interest. Link between maternal inflammation and preeclampsia is not well established. Furthermore, whether cigarette smoke promotes inflammation and also promotes severity of preeclampsia remains an open question. We herein investigated correlation of established inflammation signatures in the plasma and placental tissue from cohorts of preterm preeclampsia (PPE) and preterm pregnancies (control) with or without smoking history. Besides confirming increased levels of Flt1 and Eng in preeclampsia, we also observed an increase in various mediators of maternal inflammation in women with PPE compared to preterm cohort. Increased IL-6, IL-35, and TNF-*α* and reduced IL-10 in serum and higher MMP-12, TLR4, HMGB-1, and iNOS and lower Foxp3, CD56 transcripts in placental tissues of PPE compared to preterm pregnancies indicate an association of preterm preeclampsia with stark imbalance in maternal immune system and signatures of inflammation. Smoker PPE cohorts showed highest inflammatory signatures including statistically significant increase for many signatures compared to other cohorts. Together, these results provide evidence for association of inflammation with PPE and strong correlation of smoking with inflammatory signatures in PPE.

## 1. Introduction

Preeclampsia is a pregnancy disorder that is characterized by onset of new hypertension, proteinuria, and damage to vital organs such as kidney and liver [1]. Symptoms of preeclampsia are displayed typically after 20 weeks in gestation in about 2–8% of all pregnancies, presenting a major healthcare challenge with substantial morbidity and mortality in the mother and the newborns [1]. Based on the gestational age at diagnosis, preeclampsia is subclassified into two categories, viz., preterm preeclampsia (PPE) (before 37 weeks in gestation) and term preeclampsia (after 37 weeks in gestation) [2]. Preterm preeclampsia causes relatively more severe complications for both mother and newborn compared to term preeclampsia which shows relatively mild complications [2].

Both preterm and term preeclampsia are diagnosed with common symptoms (high blood pressure, proteinuria, and damage to vital organs), and placental deformity is unequivocally established in both [3]. However, triggers and diseases escalating events that take place during placental deformity are not fully understood. It is unclear and highly debatable whether maternal inflammatory state could be a trigger. However, growing evidence suggests that state of maternal inflammation could *affect* disease progression/promotion (worsening placental pathology) where immune cell imbalance in placental tissue, proinflammatory cytokine milieu, and higher oxidative stress collectively induce antiangiogenic factors in the placental tissue [4]. Placental ischemia may occur because of shallow trophoblast invasion which is associated with an immune imbalance where proinflammatory effector T cells are increased and Foxp3+ T regulatory cells (Tregs) are decreased. Regulatory natural killer (NK) cells are also known to exist in significant frequency in placenta and provide a local tolerance to fetus in healthy pregnancies. Hence, reduction in these cells once again can cause overt alloreactive responses towards fetal antigens. This imbalance can lead to chronic inflammation characterized by oxidative stress, proinflammatory cytokine release, and production of autoantibodies [5]. Proinflammatory cytokines and reactive oxygen/nitrogen species can also induce endothelial cell damage that leads to hypertension and systemic inflammation [6]. A recent study showed the role of BMP-4 in sEng-mediated perturbation of TGF-*β* signaling and a potential role of this cascade in initiation of hypertension [7]. Comprehensive profiling of gene and protein expressions between controls and preeclampsia tissues could help unravel the paradox and provide hints for the mediators of preeclampsia. In a similar attempt, a study analyzed the global protein profiles of placental tissue from preeclampsia compared to placental tissue of control cohort reinforcing several known as well as revealing new proteins that could be potentially involved [8].

In this study, we examined the clinical samples from preterm pregnancies and preterm preeclampsia (PPE) both at mRNA and protein levels to understand the role and association of maternal immune system and state of inflammation in affecting the fate and severity of preterm preeclampsia in patients. Inflammation can potentially lead to production of antiangiogenic factors resulting in abnormal angiogenesis in placenta as well as endothelial cell damage and hypertension [7]. The focus on PPE was due to its higher clinical impact in terms of disease severity. As previously reported, we observed the antiangiogenic factors sFlt1 and sEng overexpressed in placental tissue of PPE as compared to the expression in placental tissues of preterm pregnancy. To understand the role of maternal immune system and cigarette smoke-induced chronic inflammation, in promoting pathophysiology of preeclampsia, we performed the expression analysis of various immune mediators in serum and placenta tissue from cohorts with PPE and preterm control pregnancies in smoker and nonsmoker subsets.

We found increased levels of proinflammatory cytokines IL-6, IL-35, and TNF-*α* and reduced IL-10 in serum and higher MMP-12, TLR4, HMGB-1, and iNOS and lower Foxp3, CD56 transcripts in placental tissues of PPE compared to control preterm pregnancies regardless of smoking history. However, smoker PPE subgroup showed significantly increased antiangiogenic factors sFlt1 and sEng and IL-6 and BMP-4 compared to nonsmoker PPE subgroup and control preterm cohorts. Similarly, smoker PPE subgroup showed significantly increased transcripts of CD4, CD8, CD45, and HMGB-1 compared to nonsmoker PPE subgroup and control preterm cohorts. These results collectively provide correlative evidence for role of maternal and smoking-induced inflammation in promoting a feed forward loop that further promotes inflammation, dysfunctional angiogenesis, and possibly BMP-4-mediated hypertension in pathophysiology of PPE.

## 2. Materials and Methods

### 2.1. Patient Enrollment

#### 2.1.1. Total of 108 Pregnant Women Were Enrolled in This Study

These women were treated at the Department of Gynecology and Obstetrics, Affiliated Hospital of Xuzhou Medical University, China, between July, 2016 and June, 2020. The demographics as well as clinical characteristics of the patients enrolled in the present study are presented in Table 1. Cigarette smoking history was also recorded. *Women who had smoked 3 or more cigarettes a day at least for the period of 6* months *in the past 5* years *were considered smokers.* These pregnant women were divided into two groups based on the gestational age: (1) preterm preeclampsia (<37 weeks; *n* = 60) and (2) preterm controls (<37 weeks; *n* = 48). In the preterm control group, we only selected placenta previa patients. They delivered neonates with birth weight appropriate for gestational age and had no fetal and maternal complications or symptoms of perinatal infections. Only the patients delivered by elective cesarean section before the onset of labor were enrolled in this study. Patients with complicated pregnancies such as with genetic disorders or history of cardiovascular and renal disease were also excluded. Diagnosis was based on modified American College of Obstetricians and Gynecologists criteria [9]. Preterm preeclampsia was defined by high blood pressure (blood pressure ≥ 140/90 mmHg) and proteinuria (urine dipstick with ≥2+ protein at presentation or 24 − hour urine protein ≥ 300 mg/d).

Adverse maternal outcome also included increased alanine aminotransferase (ALT ≥ 80 U/L), reduced platelet counts (≤100 × 109/L), acute renal failure, placental abruption, intravascular coagulation, cerebral hemorrhage, pulmonary edema, eclampsia, or maternal death [10, 11]. The adverse outcomes for fetal/neonatal in the PPE group included iatrogenic delivery reported by the primary obstetrician demonstrating hypertensive complications of pregnancy, low birth weight (≤10th percentile for gestational age), Doppler findings of abnormal umbilical artery, fetal, or neonatal death [10, 11]. The research protocols followed all guidelines and were approved by the Ethics Committee of Xuzhou Medical University.

### 2.2. Placental Tissue and Blood Sampling

In total, 108 women allocated into two groups, 60 with preterm preeclampsia and 48 controls of the preterm preeclampsia, participated in the study. All participants volunteered to participate in this study and signed an informed consent form. The study was approved by the Local Ethical Committee. Placental tissues were collected aseptically within 10 minutes of cesarean sections. Sites of infarction, hemorrhage, and calcification were excluded. Placental tissues (1 cm × 1 cm × 1 cm) were collected from proximal to maternal sides of the placenta under sterile condition. Specimens were rinsed with 1X sterile phosphate buffer saline (PBS) and snap frozen in liquid nitrogen and stored in tissue bank. Maternal blood (5 mL) was collected in fasting state, allowed to clot and centrifuged for 10 min. Serum samples were collected and frozen and stored at -80 C until the day of the analysis. Of the 60 women with preterm preeclampsia, 42 women were classified as having severe preeclampsia and 18 had mild preeclampsia. Maternal age was similar in both groups as well as control preterm pregnant women cohort.

### 2.3. Protein Level Analysis by Western Blotting and ELISA

#### 2.3.1. Western Blotting

Total protein was extracted from the 108 fresh frozen placental samples. Briefly, 1 mL of cell lysis buffer (Cell Signaling Technology, Danvers, MA) containing protease and phosphatase inhibitor cocktail (Roche Applied Science, Indianapolis, IN) was added to tissue samples, and the lysates were sonicated twice for 15 seconds in ice and then centrifuged at 12,000 rpm for 30 min at 4°C to remove debris. The protein concentrations were quantified using BCA Protein Assay kit (PIERCE, Rockford, IL). The lysates containing 100 *μ*g of total proteins were separated on 10% SDS-polyacrylamide gelsat and transferred to nitrocellulose membrane. The nitrocellulose membranes were blocked for 1 hour with blocking solution containing 4% BSA in 1× TBST containing 20 mM Tris-HCl (pH 7.5), 100 mM NaCl, and 0.1% Tween-20. The desired proteins were then probed using specific primary antibodies overnight. After 3× wash with 1× TBST, secondary antibodies conjugated with horseradish peroxidase were added for 1 hour at room temperature. After 3× wash with 1× TBST, HRP substrate was added to visualize and record signal intensities by the chemiluminescence (Western Blotting Kit, Santa Cruz Biotechnology, CA, USA). To monitor equal loading of protein, Western blotting using antibody against *β*-actin was performed for each experiment as shown in lower panels.

### 2.4. ELISA

Serum from the cohorts was used for analysis of the proinflammatory cytokines. Commercial ELISA kits were used to measure concentration of IL-6, IL1*β*, IL-10, IL-35, and TNF*α* (R&D Systems, Minneapolis, MN, USA). BMP-4 from human sera was measured using the Human BMP-4 Quantikine ELISA Kit (DBP400; R&D Systems). Standard curve was developed according to the manufacturer instructions. The values obtained were expressed either as relative absorbance or as pg/ml. Manufacturer's instructions were followed for analysis and quantification of the specific cytokines.

### 2.5. RNA Expression Analysis by Quantitative Real-Time Polymerase Chain Reaction (qRT-PCR)

Total RNA from placental tissues was isolated using TRIzol followed by RNase mini prep kit from Qiagen. The RNA was treated with DNase using Turbo DNAse kit (Ambion). For quantitative real-time PCR, 1 *μ*g total RNA was reverse transcribed in 50 *μ*l reaction using TaqMan reverse transcription reagents (Applied Biosystems) using random hexamer primers. A total of 2 *μ*l cDNA and the 1 *μ*M real-time PCR primers were used in a final 20 *μ*l PCR with “power SYBR-green master mix” (Applied Biosystems). The primers for these real-time PCR assays were purchased from Real Time Primers, LLC (Elkins Park, PA). The primer sequences will be provided upon request. Real-time PCR was performed in Bio-Rad CFX-96 Real Time System. Expression of the target genes was normalized to housekeeping gene *β*-actin and was displayed as fold change relative to control samples from preterm pregnancy cohort. Data are representative of specimens isolated from 48-60 specimen samples run in duplicate for subjects used in the study.

### 2.6. Statistical Analysis

Class comparison analysis was performed using unpaired Student's *t*-test to identify proteins differentially expressed between groups. Bivariate Spearman correlation test was used to analyze the correlation between differentially expressed proteins and preterm preeclampsia severity. Proportional data were analyzed using one-way ANOVA, and significant difference between groups was determined by Tukey's test. All the assays were independently replicated at least three times, and the data were presented as mean ± SEM. Significant difference was accepted at *p* value ≤0.05 *which is denoted by*^∗^.

## 3. Results

### 3.1. Clinical Characteristics

Table 1 summarizes the clinical parameters and characteristics of the 108 pregnant women that participated in this study. As expected, women with PPE showed new onset of hypertension along with manifesting a series of other clinical symptoms described in Table 1. The gestational age at delivery for preterm preeclampsia women was slightly lower than preterm control group. Eight out of 48 women in the preterm control group had smoking history (as defined by women who had smoked *more than 3 cigarettes a day for at least 6* months in the past 5 years) whereas 18 out of 60 women in PPE cohort had smoking history. Neonatal birth weight and placental weight were also significantly lower in the PPE group compared to the control preterm group where 50% (30/60) infants from PPE patients were small for their gestational age. However, ~72% (13/18) infants from smoker PPE patients were small for their gestational age. Adverse outcomes (as described in the methods sections) were more severe and occurred in 70% of women with total PPE (*n* = 60) as opposed to 83.3% (15/18) of women with smoker PPE women.

### 3.2. Increased Expression Levels of Flt1 and Eng Proteins in Placental Tissue and Sera in Preterm Preeclampsia

**Figure 1:**
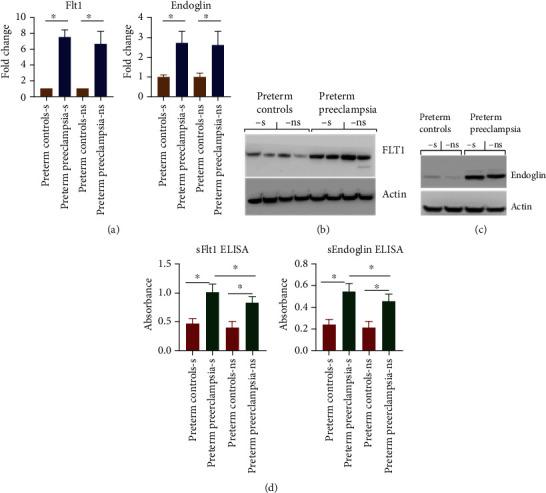
Expression of Flt1 and endoglin in placental tissue and sera of PPE compared to control cohort. (a) mRNA levels of the Flt1 and Eng transcripts analyzed by real-time polymerase chain reaction (RT-PCR). mRNA abundance quantified with RT-PCR represents quantifications from both control preterm and preterm preeclampsia cohorts with/out smoking history. (b, c) Analysis of Flt1 and Eng at protein levels in placental tissue of preterm preeclampsia compared to control cohort with/out smoking history by Western blotting. Representative Western blot image is shown here. (d) Analysis of the soluble form of Flt1 and Eng in sera of PPE compared to control cohort with/out smoking history by ELISA. Sera were diluted 1 : 10 and quantified with ELISA. The figure is cumulative results of *n* = 8‐30 depending upon the subgroup. s denotes smoker, and ns denotes nonsmoker. ^∗^ represents statistically different with *p* value <0.05.

Total mRNA and tissue lysates were prepared from the placental tissue specimen. Analysis of Flt1 and Eng at the transcript level using real-time polymerase chain reaction (RT-PCR) showed increased expression of both Flt1 and Eng genes at mRNA levels in placental tissues of PPE as compared to the mRNA expression level in placenta of preterm pregnancy controls regardless of smoking history (Figure 1(a)). Similarly, Flt1 and Eng proteins were also elevated in placental tissues of PPE as compared to the level in placenta of preterm pregnancy controls regardless of smoking history as analyzed by Western blotting (Figures 1(b) and 1(c)). However, soluble circulating levels of soluble (s)Flt1 and sEng in serum samples were significantly increased in smoker PPE subset as compared to nonsmoker PPE subgroup and control preterm cohorts (Figure 1(d)).

### 3.3. Immune Cell Imbalance in Placental Tissue in Preterm Preeclampsia

**Figure 2:**
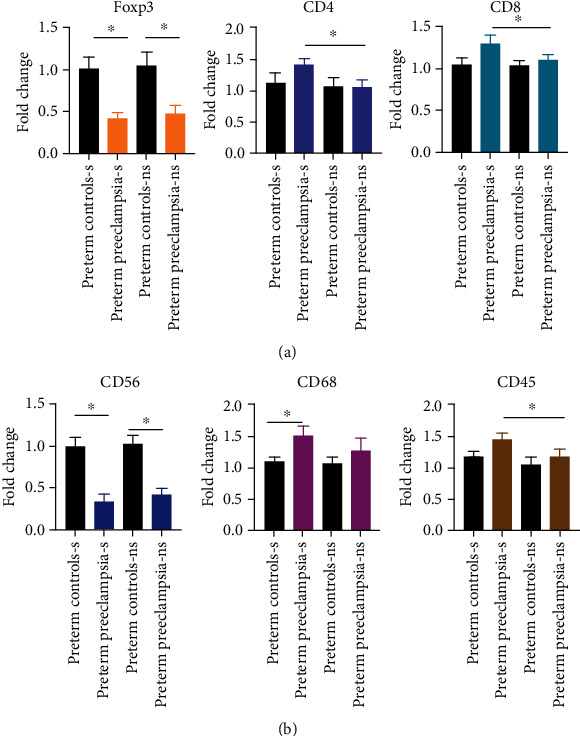
Immune cell analysis in placental tissue specimens from PPE vs. preterm controls with/out smoking history. mRNA levels of the human Foxp3, CD4, CD8, CD56, CD68, CD45, transcripts were analyzed in placental tissue by real-time polymerase chain reaction (RT-PCR) in PPE vs. preterm controls with/out smoking history. 1 *μ*g total RNA was reverse transcribed in 20 *μ*l reaction. A total of 2 *μ*l cDNA and the 1 *μ*M real-time PCR primers purchased from Real Time Primers, LLC (Elkins Park, PA) were used for amplification in Bio-Rad CFX-96 Real Time System. Expression of the target genes was normalized to *β*-actin and displayed as fold change relative to the preterm control sample. Data are representative of specimens isolated from 8-30 specimen samples belonging to subjected used in the study. s denotes smoker, and ns denotes nonsmoker. ^∗^ represents statistically different with *p* value <0.05.

It is proposed that CD4+Foxp3+T regulatory cells and regulatory NK cells are prevalent in placental tissues and provide local tolerance to placental tissue and fetus by limiting chronic inflammation mediated by CD4+effector T cells [12]. In this context, the ratio of Foxp3+T regulatory cells to effector CD4 and CD8 T cells could be an indicator of chronic inflammation at the local site. To address this and state of overall immune landscape, mRNA expression of Foxp3, CD4/8, CD45, CD56, and CD68 transcripts was analyzed by RT-PCR in the placental tissue of PPE as well as the preterm controls with/out smoking history. Foxp3 expression was found to be significantly lower in the placental tissue of PPE compared to preterm controls regardless of smoking history (Figure 2(a)), whereas both CD4 and CD8 expressions were found to be significantly higher in smoking PPE subgroup than other cohorts suggesting an imbalance between T regulatory cells and T effector cells (Figure 2(a)). Significantly reduced NK cells (CD56 transcripts) in PPE cohort compared to preterm cohort were observed regardless of smoking history (Figure 2(b)). A significant increase was also observed in macrophages (CD68 transcripts) in smoker PPE subgroups compared to smoker preterm control (Figure 2(b)), whereas CD45 transcripts (overall immune cell infiltrate) were significantly high in smoker PPE subset as compared to all other subgroups (Figure 2(b)) suggesting an overall increase in infiltration of immune cells in placental tissue. Together, these transcript analyses reveal an overall imbalance towards activated maternal immune responses in placental tissue and confirm a tight association of inflammation with smoking in preterm preeclampsia.

### 3.4. Expression of MMP-12, Reactive Nitrogen Species, and Danger Signals

**Figure 3:**
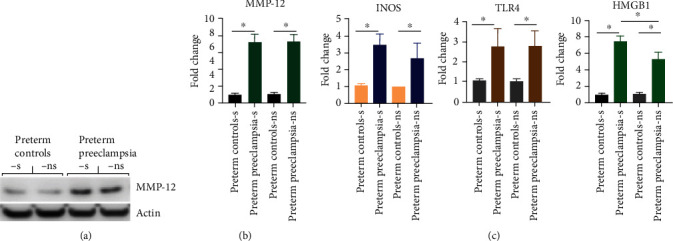
Analysis of MMP-12, iNOS, TLR4, and HMGB-1 expressions from PPE vs. preterm controls with/out smoking history. (a) Analysis of MMP-12 proteins in the placental tissues of preterm preeclampsia compared to preterm pregnancy cohort with/out smoking history by Western blotting. MMP-12 expression data shown in the WB image is a representative of specimens isolated from 8-30 specimen samples belonging to subjected used in the study. (b, c) mRNA levels of the human MMP-12, iNOS, TLR4, and HMGB-1 transcripts were analyzed in placental tissue by real-time polymerase chain reaction (RT-PCR). 1 *μ*g total RNA was reverse transcribed in 20 *μ*l reaction. A total of 2 *μ*l cDNA and the 1 *μ*M real-time PCR primers purchased from Real Time Primers, LLC (Elkins Park, PA) were used for amplification in Bio-Rad CFX-96 Real Time System. Expression of the target genes was normalized to *β*-actin and displayed as fold change relative to the preterm control sample. Data are representative of specimens isolated from 8-30 specimen samples belonging to subjected used in the study. s denotes smoker, and ns denotes nonsmoker. ^∗^ represents statistically different with *p* value <0.05.

To understand the upstream mechanism that leads to upregulation of sFlt1 and sEng proteins in placental tissues and possibly in peripheral blood, we focused on macrophage-associated matrix metallopeptidase 12 (MMP-12) as it can cleave the cell surface proteins [13]. Both at mRNA and protein levels, expression of MMP-12 was found to be dramatically increased in the placental tissues of PPE cohorts as compared to their levels in placental tissues of preterm controls regardless of smoking history (Figure 3(a)). Analysis of expression of several genes that are crucial to drive inflammatory responses revealed dramatic increase in inducible nitric oxide synthase (iNOS: a gene involved in NO production) and toll-like receptor 4 (TLR4; a sensing receptor for inflammation) in antigen presenting cells such as macrophages in PPE compared to preterm pregnancy cohort regardless of smoking history (Figure 3(b)). However, TLR4 endogenous ligand high mobility group box 1 (HMGB-1) was significantly upregulated in smoker PPE subset compared to other subsets (Figure 3(b)). These results suggest that smoking could be involved in promotion of HMGB-1: TLR4-mediated promotion of inflammation in PPE.

### 3.5. Bone Morphogenic Protein-4 (BMP-4) and Pro-/Anti-Inflammatory Cytokines in the Sera of Preterm Preeclampsia with Smoking History

**Figure 4:**
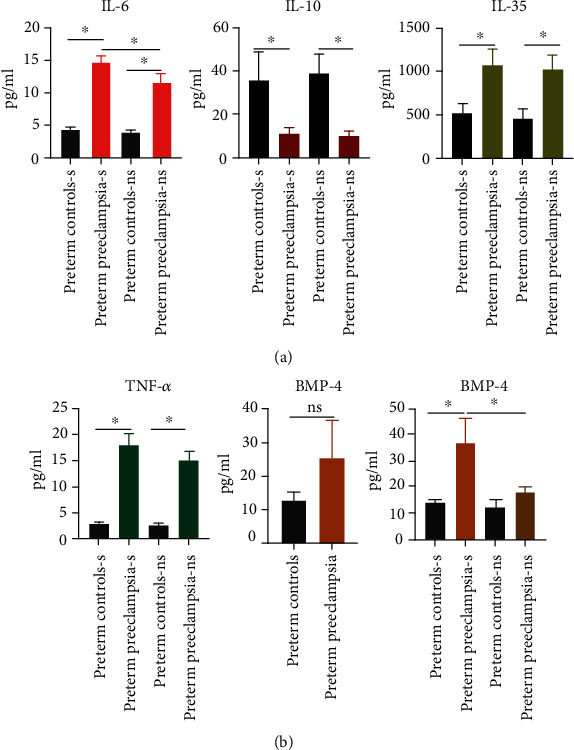
Evaluation of pro-/anti-inflammatory cytokines and BMP-4 levels from PPE vs. preterm controls with/out smoking history. (a, b) Analysis of proinflammatory cytokines TNF-*α*, IL-1B, IL-6, IL-35, and IL-10 in sera of preterm preeclampsia compared to control preterm cohort by ELISA. The serum samples were diluted in sample buffer as per manufacturer's recommendation, and manufacturer's instructions were followed for analysis and quantification of the specific cytokines. ELISA results shown here are from 8-30 specimen samples belonging to subjected used in the study. (b) Analysis of BMP-4 in sera of preterm preeclampsia compared to control cohort by ELISA. s denotes smoker, and ns denotes nonsmoker. ^∗^ represents statistically different with *p* value <0.05.

**Figure 5:**
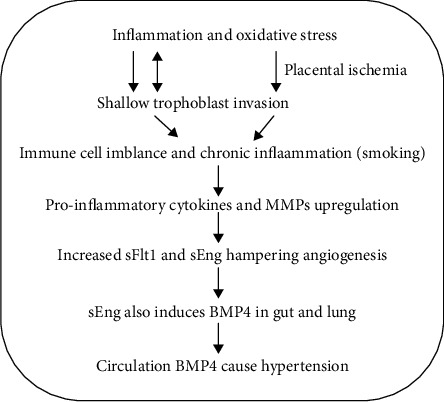
Schematic diagram showing the potential mechanisms of preeclampsia progression and proposed role of chronic inflammation in onset and promotion of preeclampsia.

Analysis of proinflammatory cytokines revealed overall increase in TNF-*α*, IL-6, and IL-35 and a decrease in IL-10 in the sera than of PPE compared to preterm pregnancy regardless of smoking history (Figures 4(a) and 4(b)). However, IL-6 was significantly higher in smoker PPE as compared to nonsmoker PPE (Figure 4(b)). A recent study elegantly demonstrated a potential role of BMP-4 in sEng-mediated perturbation of TGF-*β* signaling that leads to hypertension [7]. BMP-4 levels were analyzed in sera of PPE cohorts as compared to preterm pregnancy. As shown in Figure 4(a), the levels of BMP-4 were higher in sera of PPE cohorts as compared to preterm control cohort. Although BMP-4 levels were somewhat variable and not statistically significant, when BMP-4 levels were compared between the two cohorts that shared smoking history, BMP-4 levels in the sera of smoker PPE cohort were significantly higher than BMP-4 levels in the sera of smoker preterm cohort or nonsmoker cohorts of either groups (Figure 4(a)). Together, these results suggest that smoking along with enhanced maternal inflammation in PPE may help induce production of higher BMP-4 in this cohort. The potential involvement of BMP-4 and inflammatory mediators and crosstalk in context of other factors involved in preterm preeclampsia are depicted in the sketch diagram (Figure 5).

## 4. Discussions

Preeclampsia, in particular preterm preeclampsia, presents a major health challenge and can lead to catastrophic outcome for the mother and neonate. On clinical side, onset of hypertension, proteinuria, and multivital organ damage are primary symptoms for diagnosis. Perturbed angiogenesis in placental tissue and role of antiangiogenic molecules such as soluble or cleaved forms of Flt1 and Eng have been demonstrated over a decade [14, 15]. However, the triggers that initiate and the promoter events that amplify severity in preeclampsia in pregnant women are poorly understood. Some evidence suggests a plausible role of chronic inflammation or inflammatory cytokines that could lead to induction of MMPs. MMPs are involved in proteolytic cleavage of cell surface molecules such as Flt1 and Eng that are distinctly associated as antiangiogenic mediators of preeclampsia [14, 15]. Similarly, downstream connection of sFlt1 and sEng to mediators that could cause hypertension is not fully demonstrated. Cigarette smoking is long associated with lung cancers and promotion of lung cancer by promoting inflammation. Whether smoking-mediated inflammation promotes severity of PPE also remains to be well established.

In this study, we used clinical samples for evaluating the paradigm that whether state of maternal immune system and cigarette smoke-induced inflammation have strong association with pathophysiology and its severity in preterm preeclampsia. We present correlative evidence here that indicates that chronic inflammation may play a critical role in pathology of preeclampsia. We show that cytokines TNF-*α*, IL-6, and IL-35 are elevated, and IL-10 is reduced in the sera of preterm preeclampsia cohort as compared to control preterm cohort, suggesting a role of chronic inflammation in disease pathology. Also, IL-6 was found to be significantly higher in smoker PPE compared to nonsmoker PPE suggesting a role of smoking in enhanced inflammation.

Mounting evidence suggests that inflammation could be a promotor of events that leads to amplification of vicious cycle to adverse pathology in preeclampsia, and controlling inflammatory responses could be a potential avenue for improving the outcome in patients with preeclampsia. In support of this notion, cyclosporin A (CsA), an immunosuppressant, improved clinical characteristics of preeclampsia and suppressed inflammation in a lipopolysaccharide- (LPS-) induced preeclampsia rat model [16]. Another study showed that treatment with low-dose aspirin in women at high risk for preterm preeclampsia resulted in a lower incidence of this diagnosis than placebo [17]. Given the anti-inflammatory properties of aspirin, one potential mechanism by which aspirin may reduce preterm preeclampsia risk is through inhibition of chronic inflammation. Chronic inflammation is characterized by aberrant long-term expression of circulating inflammatory factors such as cytokines, danger signals, and growth factors. We observed increased cytokines and HMGB-1 suggesting ongoing inflammation at local and systemic compartments of maternal immune system. Another recent report observed that preterm preeclampsia cohorts had more subjects with smoking history as compared to term preeclampsia patients [18]. Cigarette smoke (CS) causes chronic inflammation, lung and vascular disease inflammation, and release of cytokines [18]. In current study, we also observed that higher fraction (18/60) of preterm preeclampsia women were smokers as opposed to 8/48 in preterm cohort. Many inflammatory signatures (sFlt1, sEng, effector T cell markers, HMGB-1, BMP-4, and IL-6) were all significantly increased in smoker PPE as compared to nonsmoker PPE. These studies along with our findings show a role of smoking and smoking-induced inflammation as a potential enhancer of this pathology.

We herein reported increased IL-6, IL-35, and TNF-*α* that can induce myeloid cell MMP-12 which could exacerbate the disease by cleavage of membrane, the Flt1 and Eng [19]. Although other MMPs are known to be dysregulated in preterm preeclampsia [20, 21], the focus on MMP-12 was due to its critical role in inflammation-mediated activity [22, 23]. We show enhanced MMP-12 expression in the placenta tissue of preterm preeclampsia reinforcing this notion. Increased Flt1 and Eng transcripts as well as proteins were observed in placenta of preterm preeclampsia that can dysregulate angiogenesis leaving placental tissue and embryonic tissues in state of hypoxia [24]. Hypoxia may lead to further disruption of angiogenesis due to damage of vascular endothelium which is also known to cause hypertension and systemic inflammation [7, 25]. This completes the feed forward loop for deteriorating pathology seen in the preeclampsia (Figure 5). One paradoxical finding was the observation of increased Flt1 and Eng transcripts by RT-PCR as proteins are cleaved and soluble proteins primarily amount for pathology. It is possible that increased transcription of Flt1 and Eng may be a result of compensatory mechanism that is engaged to compensate the protein expression of membrane bound Flt1 and Eng.

A recent study demonstrated the important role of BMP-4 in perturbation of TGF-*β* signaling and induction of hypertension [7]. We herein provide an independent evaluation and confirm the increased levels of BMP-4 in the sera of preterm preeclampsia patients (Figure 4(a)). Most interestingly when BMP-4 levels were further divided into smokers and nonsmokers in both cohorts, BMP-4 levels in smoker PPE were significantly higher than smoker preterm controls suggesting a role of smoking-mediated inflammation in BMP-4 increase. Proinflammatory cytokines TNF and IL-35 were also slightly higher in smoker PPE than nonsmoker PPE subgroup suggesting a trend that indicates synergy between smoking and maternal inflammation leading to advanced stage PPE.

Based on our limited observations and recent literature, we propose a theoretical model (Figure 5) where chronic inflammation, perhaps in combination with other factors (smoking or other inflammatory agents), during pregnancy can potentially trigger elevated MMP-12 and proinflammatory cytokine expressions that leads to increased sFlt1 and sEng. sFlt1 hampers angiogenesis by sequestering VEGF whereas sEng also directly induces BMP-4 as a result of its signaling in gut and lung. BMP-4 has been shown to be a mediator of hypertension (Figure 5). These two mechanisms, i.e., dysfunctional angiogenesis and endothelial damage along with hypertension, further increase the inflammation for a self-feeding loop that exacerbates the disease pathology. It is likely that pathophysiology of the preeclampsia is far more complex than presence of chronic inflammation and smoking-mediated increase of inflammation. Other risk factors could be metabolic deficiencies, genetics, and cardiovascular health. It is plausible that all these factors may act in concert with chronic inflammation for the development of preeclampsia. Detailed studies of these pathways may provide further insights that could lead to discovery of better biomarkers as well as therapeutics for better outcome in preeclampsia.

## Figures and Tables

**Table 1:** Demographic and clinical characteristics of the study groups.

Characteristics	Preterm controls (*n* = 48)	Preterm preeclampsia (*n* = 60)
Women		
Age (yr)	26.6 ± 5.2	26 ± 4.9
Weight (kg)	70 ± 2.6	76.8 ± 4.9
Height (inch)	161 ± 3.2	161.6 ± 2.8
Body mass index	27.2 ± 1.2	30.1 ± 1.7
Nulliparous—no. (%)	31.7 (88.5)	56 (81.3)
Highest SBP at triage (mmHg)	122 ± 12.4	176 ± 18.8
Highest DBP at triage (mmHg)	80.2 ± 11.1	115 ± 16.3
Gestational age at triage (wk)	33.4 ± 3.1	33.2 ± 3.0
Gestational age at delivery (wk)	33.9 ± 2.3	33.6 ± 2.6
Proteinuria—no. (%)	0 (0%)	30 (100%)
ALT at triage (U/L)	23.6 ± 26.8	48.7 ± 96.8
Creatinine at triage (*μ*mol/L)	41.3 ± 6.3	78.2 ± 29.2
Uric acid at triage (*μ*mol/L)	160 ± 16.5	365.8 ± 90.2
Platelet count at triage (6109/L)	234.6 ± 48.2	168.3 ± 70.6
Subjects with smoking history	8/48	18/60
Infants		
Birth weight (g)	2336.2 ± 495.5	1920.6 ± 620.2
Delivery, 37 wk—no. (%)	24 (100%)	30 (100%)
Placenta		
Placenta weight (g)	466.4 ± 95.5	402.1 ± 82.3

## Data Availability

The data used to support the findings of this study are included within the article.
